# Diversity of Red Yeasts in Various Regions and Environments of Poland and Biotechnological Potential of the Isolated Strains

**DOI:** 10.1007/s12010-023-04705-5

**Published:** 2023-08-30

**Authors:** Anna M. Kot, Wioletta Sęk, Marek Kieliszek, Stanisław Błażejak, Katarzyna Pobiega, Rita Brzezińska

**Affiliations:** 1 Department of Food Biotechnology and Microbiology, Institute of Food Sciences, Warsaw University of Life Sciences, Nowoursynowska 159C, 02-776 Warsaw, Poland; 2 Department of Chemistry, Institute of Food Sciences, Warsaw University of Life Sciences, Nowoursynowska 159C, 02-776 Warsaw, Poland

**Keywords:** Red yeast, Carotenoid pigments, Torulene, Torularhodin, *Rhodotorula*

## Abstract

Due to the growing demand for natural carotenoids, researchers have been searching for strains that are capable of efficient synthesis of these compounds. This study tested 114 red yeast strains collected from various natural environments and food specimens in Poland. The strains were isolated by their ability to produce red or yellow pigments in rich nutrient media. According to potential industrial significance of the carotenoids, both their total production and share of individual carotenoids (β-carotene, γ-carotene, torulene, and torularhodin) were analyzed. The total content of carotenoid pigments in the yeast dry matter ranged from 13.88 to 406.50 µg/g, and the percentages of individual carotenoids highly varied among the strains. Most of the yeast isolates synthesized torulene at the highest amount. Among the studied strains, isolates with a total carotenoid content in biomass greater than 200 µg/g and those containing more than 60% torularhodin were selected for identification (48 strains). The identified strains belonged to six genera: *Rhodotorula*, *Sporidiobolus*, *Sporobolomyces*, *Buckleyzyma*, *Cystofilobasidium*, and *Erythrobasidium*. The largest number of isolates belonged to *Rhodotorula babjevae* (18), *Rhodotorula mucilaginosa* (7), *Sporidiobolus pararoseus* (4), and *Rhodotorula glutinis* (4).

## Introduction

Carotenoids are a group of compounds widely used in pharmaceutical, chemical, food, and feed industries due to their ability to act as vitamin A precursors as well as their coloring, antioxidant, and potentially disease-inhibiting properties. Many species of yeasts classified under Basidiomycota, which grow as pigmented colonies and are therefore known as “red yeast,” are capable of producing carotenoids. These include genera such as *Rhodotorula*, *Rhodosporidium*, *Sporobolomyces*, *Sporidiobolus*, *Xanthophyllomyces*, and *Cystofilobasidium*. These yeasts synthesize compounds including astaxanthin, β-carotene, torulene, or torularhodin, which give their colonies a characteristic orange, salmon, pink, or red color [1].

Humans and animals cannot synthesize carotenoids; therefore, these compounds must be supplied in the diet. Because various microorganisms can synthesize several important carotenoids, they are recognized as a potential natural source of these compounds. Biotechnologically produced carotenoids are an alternative to synthetic compounds, which, according to studies, may have negative effects on health, and even increase the risk of cancer when consumed in excess [2]. Currently, the microalga *Haematococcus pluvialis* and the mold *Blakeslea trispora* are mainly used for the biotechnological production of carotenoids (astaxanthin and β-carotene, respectively) [3].

β-Carotene belongs to the group of nonoxidized carotenoids and is one of the widely studied pigments. It is composed of two β-ionone rings connected together by a polyene chain consisting of nine conjugated double bonds [4]. β-Carotene is a strong antioxidant that can protect cells against damage by light and oxygen. It exhibits the highest provitamin A activity compared to other carotenoids. One molecule of β-carotene is converted into two molecules of vitamin A, and thus, this compound is considered as an important source of vitamin A for humans. β-Carotene is a fat-soluble, red-orange pigment used not only in the food industry but also as a feed additive and in the cosmetic industry [5]. Some yeasts of the genera *Rhodotorula*, *Sporobolomyces*, and *Sporidiobolus* are efficient producers of this compound [3].

Torulene and torularhodin are fungal carotenoids. Torulene contains one β-ionone ring attached to a polyene chain. It also has 13 double bonds, and thus exhibits stronger antioxidant properties than β-carotene. As β-ionone ring is the backbone of vitamin A, torulene can act as a precursor of this vitamin. This orange pigment is predominantly produced by yeasts of the genera *Rhodotorula*, *Sporidiobolus*, and *Sporobolomyces* [6].

Torularhodin is a red carotenoid produced mainly by *Rhodotorula* and *Sporobolomyces* yeasts. Due to the presence of a carboxyl group at the end of the polyene chain, this compound is classified under the group of xanthophylls. Torularhodin is produced as a result of the oxidation of torulene. It is a potent antioxidant that helps to stabilize cell membranes under stressful conditions. Torularhodin can more effectively quench singlet oxygen than β-carotene, remove free radicals, and inhibit the formation of lipid peroxides, protecting cells, among others, against tumors. Moreover, this compound exhibits antimicrobial activity against both Gram-positive and Gram-negative bacteria [7].

Red yeasts are widespread in nature. These organisms can be found in flowers, air, fruit, and leaves, and can contaminate foods such as Greek yogurt, cream, and ground pork. Red yeasts have previously been isolated from the natural environment on all continents [8], including extreme environments such as Antarctic glaciers [9, 10].

During the study, 408 specimens were sampled from various natural environments and food products in Poland. The goals of research were to study distribution and diversity of red yeasts and, as well, to study production of carotenoids by the isolates for their possible industrial application.

## Materials and Methods

### Isolation of Red Yeast

**Fig. 1:**
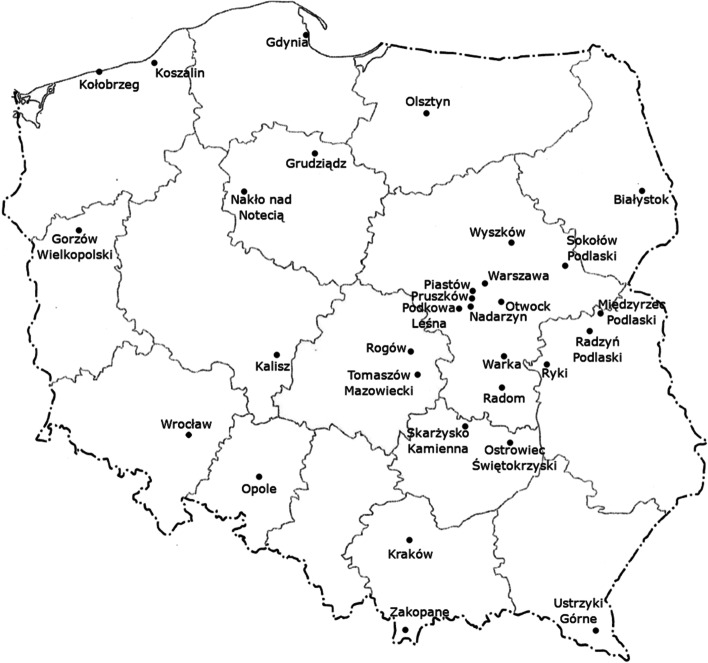
Cities in Poland where samples were taken for the isolation of red yeasts

Yeast isolation was carried out from February 2019 to September 2020 in Poland (Fig. 1). 408 specimens were sampled from various sources for isolation of red yeasts: air (124), flowers (78), tree leaves and bark (56), fruit (56), tree slime fluxes (31), propolis (15), cereals (13), soil (9), vegetables (8), bee pollen (6), cheese (4), meat (3), bee bread (2), the Baltic Sea water (2), and chocolate (1). For the isolation of yeast from the air, Petri dishes containing the YPD medium were left open for 15 min and then incubated at 22℃ for 96 h. Other materials were placed in sterile bags or containers for their transportation to the research laboratory. Samples that could not be immediately transported to the laboratory after collection were stored at − 18℃. The samples were transferred to 100 mL of liquid YPD medium (20 g/L glucose, 20 g/L peptone, 10 g/L yeast extract, BTL, Poland) and incubated at 22℃ for 48 h. After incubation, the proliferated material was inoculated into Sabouraud agar with chloramphenicol (BTL, Poland), and then incubated again at 22℃ for 96 h. After incubation, orange-, red-, or pink-colored colonies were observed and were reductively transferred to Sabouraud agar with chloramphenicol for the isolation of pure yeast cultures. The obtained yeast isolates were taken by loop, transferred to 80 mL of liquid YPD medium, grown on a shaker (New Brunswick™ Innova® 44R, Eppendorf) at 140 rpm for 24 or 48 h at 22℃, and then frozen with glycerol (50%, v/v) at − 85℃.

### Culture Conditions and Media

Pure yeast cultures were streaked on YPD agar medium and incubated at 22℃ for 96 h. After incubation, a yeast colony was taken using a loop and transferred to a liquid medium (glucose 30 g/L, yeast extract 10 g/L, KH_2_PO_4_ 1.5 g/L, MgSO_4_∙7H_2_O 1 g/L, (NH_4_)_2_SO_4_ 5 g/L, CaCl_2_ 0.15 g/L, pH 5.6) in 500-mL flatbottomed flasks. The isolates were grown on a shaker (New Brunswick™ Innova® 44R, Eppendorf) at 140 rpm for 96 h at 22℃.

### Biomass Yield

To determine the biomass yield, 10 mL of yeast cultures was centrifuged (Eppendorf Centrifuge 5804R) at 10,000 rpm for 10 min. The resulting supernatant was removed, and the samples were dried at 105℃ for 24 h. The results below are presented as grams of dry matter per liter of the medium (g/L).

### Determination of Carotenoid Content by Spectrophotometric Method

To determine the carotenoid content, 1 mL of liquid yeast culture was centrifuged (10,000 rpm for 10 min), suspended in 2 mL of dimethyl sulfoxide, and 0.5 mm glass beads were added to disrupt yeast cells. The samples were shaken for 1 h at 70 rpm (Rotator Multi Bio RS-24, Biosan) to extract intracellular carotenoids. Then, 2 mL of acetone, petroleum ether (with 0.25% BHT), and 20% sodium chloride were added. The samples thus prepared were repeatedly shaken for 1 h and centrifuged at 3500 rpm for 5 min to separate the phases of the mixture. The colored ether phase was transferred to a cuvette, and its absorbance was measured in a spectrophotometer at a wavelength of 490 nm. The total carotenoid content (µg/g_d.m_.) was calculated using the formula = A_max_ × D × V/(E × W), where A_max_ is the absorbance at 490 nm, D is the sample dilution factor, V is the volume of the ether phase, E is the carotenoids extinction coefficient (0.16), and W values were calculated from the biomass yield data. If the yeast biomass remained colored after the first extraction step, the process was repeated analogously with 2 mL of DMSO [11, 12].

### Analysis of the Carotenoid Profile by HPLC

To determine the percentages of β-carotene, γ-carotene, torulene, and torularhodin in the total pool of carotenoids, the samples were extracted using the same method applied for the determination of the total content of carotenoids by spectrophotometric method. Then, petroleum ether was evaporated under a nitrogen atmosphere, and 1 mL of mobile phase (a mixture of acetonitrile, isopropanol, and ethyl acetate in a 4:4:2 volume ratio) was added. The sample was filtered through a 0.45-µm filter and separated on a C18 analytical column (Bionacom, 250 mm × 4.6 mm, 5 μm). The flow rate of the mobile phase was set at 0.7 mL/min (isocratic). Detection was performed with a UV–VIS detector at a wavelength of 490 nm. Identification of β-carotene and γ-carotene was carried out based on the retention of standards (Sigma-Aldrich, Poland). Torulene and torularhodin were identified based on the retention time of the patterns separated by thin-layer liquid chromatography [13]. The percentages of β- and γ-carotene, torulene, and torularhodin in the carotenoids mixture were determined based on the area of the peaks.

### DNA Isolation Using the Chloroform–Phenol Method

For the isolation of DNA from yeast, a colony was taken from the YPD agar and suspended in sterile water. The samples were thoroughly mixed and centrifuged at 14,000 rpm for 10 min (Mini Spin Plus, Eppendorf). Then, the supernatant was removed, 300 µL of lysis buffer was added to the biomass, and the samples were incubated at 500 rpm at 37℃ for 1 h (Thermomixer Comfort, Eppendorf). After incubation, 200 µL of TE buffer was added and each sample was mixed for 1 min. Next, 200 µL of phenol:chloroform:isoamyl alcohol (25:24:1) at pH 8.0 (Sigma-Aldrich, Poland) was added, and each tube was mixed for 1 min. The samples were centrifuged at 14,000 rpm for 10 min. The top layer was collected, and 600 µL of cold 96% ethanol was added and mixed for 30 s. The prepared samples were incubated for 30 min at − 18 °C, and centrifuged at 14,000 rpm for 10 min. The supernatant was removed, 500 µL of 70% ethanol was added, and the mixture was centrifuged again under the same conditions. Finally, ethanol was removed and the extracted DNA was resuspended in 25 µL of sterile nuclease-free water.

### DNA Amplification

The molecular method used for yeast identification was based on amplification and sequence analysis of the D1/D2 domain of the large subunit ribosomal DNA (28 S rDNA). The LSU domain was amplified using the primers NL1 (5′-GCATATCAATAAGCGGAGGAAAAG-3′) and NL4 (5′-GGTCCGTGTTTCAAGACGG-3′). The PCR mixture contained, apart from the other primers (NL1 and NL4, 20 pmol each), the same components as for the reaction with the ITS primers. The amplification conditions were as follows: initial denaturation—91℃ for 2 min; 36 cycles—denaturation at 91℃ for 1 min, hybridization at 60℃ for 1 min, and extension at 72℃ for 1 min; final extension—72℃ for 15 min. The PCR products were sent to Genomed S.A. (Warsaw, Poland) for sequencing by enzymatic synthesis (Sanger’s dideoxy). The sequences obtained in the form of a chromatogram were analyzed using the Chromas Lite program (version 2.6.6). Further analysis of sequences from each isolate was performed using BLAST from the National Center for Biotechnology Information (NCBI) with Genbank® genetic sequence database. Strain sequences were submitted to GenBank. The strains were also deposed in the Collection of Microorganisms of the Institute of Food Sciences (CMIFS) of the Warsaw University of Life Sciences.

### Morphological and Physiological Characteristics

The isolates were assessed to determine their ability to assimilate nitrates and various carbon sources (glucose, xylose, galactose, arabinose, rhamnose, fructose, maltose, sucrose, lactose, raffinose, lactic acid, tartaric acid, citric acid, glucuronic acid, methanol, ethanol, glycerol, mannitol, trehalose, xylitol, erythritol, inulin), ability to grow in the absence of vitamins, and ability to grow at 35℃. The cell shape and reproduction method were also determined. The obtained results were compared with the taxonomic keys included in “The Yeast—A Taxonomic Study” [8].

### Statistical Analysis

Pearson correlation coefficient was determined for the yield of cellular biomass, the total content of carotenoids, and the percentages of individual carotenoid fractions (β-carotene, γ-carotene, torulene, and torularhodin). The significance of differences between the mean lipid content in yeast cell biomass was assessed using the one-way analysis of variance and Tukey’s test. The significance level was set at *α* = 0.05. All analyses were performed in R Studio (version 8.9).

## Results and Discussion

### Isolation of Red Yeast

More than 130 yeast strains were isolated from the sampled specimens, and the colonies of the strains were characterized by a salmon, orange, pink, or pink-red color. Unfortunately, some of them did not survive freezing (with the addition of a cryoprotective substance) at − 85℃. As a result, a total of 114 pure cultures of red yeasts were finally obtained. A majority of the strains were isolated from air (37) and flower petals (36). Moreover, red yeast was not isolated from any tested samples of propolis (15), soil (9) and water from the Baltic Sea (2).

### Carotenoid Biosynthesis

For carotenoid biosynthesis, red yeast culture was carried out in a liquid medium at 22℃. The growth of the yeasts varied greatly, and the yield of cellular biomass ranged from 2.00 to 19.55 g/L (Table 1). The lowest yields (below 5 g/L) were obtained for A-007, A-022, A-024, A-040, A-042, A-051, and A-090 strains. The biomass yield of 56 strains ranged from 15.00 to 19.55 g/L, while that of 51 strains from 5.60 to 14.90 g/L. A comparison of the biomass yields obtained in this study with that of Chreptowicz et al. [14] showed that the yeast strains isolated here were characterized by high cellular biomass yields. The cited authors also investigated the growth and carotenoid biosynthesis capacity of new red yeast isolates and determined cellular biomass in the range of 4.5–9.7 g/L. A similar thematic scope was observed in the work of El-Banna et al. [15], who achieved a biomass yield of 9.5–11.5 g/L for the tested red yeast strains.

**Table 1:** Characteristics of isolated red yeasts and the values of their dried biomass yield, total carotenoid content in the dried biomass, and percentage of carotenoids after 96 h of cultivation

Strain no.	Source	Location	Biomass yield (g/L)	Carotenoid content in biomass (µg/g)	Percentages of individual fractions of carotenoids (%)				
					Torularhodin	Torulene	γ-Carotene	β-Carotene	Others
001	Birch sap (*Betula pendula*)	Grudziądz (53°29′02″N, 18°45′13″E)	17.45 ± 0.49	175.46 ± 3.13	9.29 ± 1.20	77.35 ± 1.49	2.63 ± 0.59	11.77 ± 0.29	0.00
002	Birch sap (*Betula pendula*)	Wyszków (52°35′34″N, 21°27′30″E)	15.43 ± 1.93	222.62 ± 6.88	26.73 ± 4.01	61.57 ± 3.63	2.22 ± 0.42	9.46 ± 0.04	0.00
003	Birch bark (*Betula pendula*)	Skarżysko-Kamienna (51°06′47″N, 20°52′17″E)	17.65 ± 0.21	106.94 ± 0.28	1.06 ± 0.24	87.51 ± 1.02	2.23 ± 0.62	9.20 ± 0.64	0.00
004	Birch bark (*Betula pendula*)	Otwock (52°06′20″N, 21°15′40″E)	18.23 ± 0.33	141.36 ± 14.84	9.32 ± 1.00	73.01 ± 1.29	6.22 ± 0.06	11.44 ± 0.35	0.00
005	Birch bark (*Betula pendula*)	Skarżysko-Kamienna (51°06′47″N, 20°52′17″E)	17.07 ± 0.38	101.84 ± 2.06	14.08 ± 1.68	81.68 ± 1.69	1.34 ± 0.15	2.89 ± 0.16	0.00
006	Birch bark (*Betula pendula*)	Skarżysko-Kamienna (51°06′47″N, 20°52′17″E)	17.10 ± 0.33	132.15 ± 8.75	4.68 ± 1.16	74.64 ± 1.96	2.25 ± 0.41	18.42 ± 1.21	0.00
007	Birch slime flux (*Betula pendula*)	Skarżysko-Kamienna (51°06′47″N, 20°52′17″E)	4.0 ± 0.71	127.70 ± 11.90	0.00	0.00	3.34 ± 1.39	46.74 ± 2.28	49.93 ± 2.28
008	Birch slime flux (*Betula pendula*)	Skarżysko-Kamienna (51°06′47″N, 20°52′17″E)	17.25 ± 0.49	180.29 ± 10.20	23.00 ± 1.33	60.30 ± 0.47	2.30 ± 0.17	12.85 ± 0.41	1.55 ± 0.28
009	Oak leaf (*Quercus* L.)	Warszawa (52°13′44″N, 21°00′59″E)	15.60 ± 0.85	223.53 ± 13.86	25.89 ± 1.36	50.06 ± 0.61	1.85 ± 0.41	22.20 ± 1.56	0.00
010	Oak leaf (*Quercus* L.)	Żyrardów (52°02′55″N, 20°26′45″E)	15.75 ± 0.07	204.75 ± 3.57	29.92 ± 0.87	16.02 ± 0.18	3.14 ± 0.01	48.43 ± 1.39	0.79 ± 0.22
011	Goatsbeard leaf (*Aruncus dioicus*)	Skarżysko-Kamienna (51°06′47″N, 20°52′17″E)	26.85 ± 1.20	139.49 ± 21.49	12.61 ± 1.94	61.90 ± 0.62	1.77 ± 0.75	23.73 ± 2.07	0.00
012	Astilbe leaf (*Astilbe* spp.)	Rogów (51°49′03″N, 19°53′11)	14.00 ± 0.57	235.22 ± 19.61	5.99 ± 1.39	59.99 ± 0.38	2.75 ± 0.04	31.28 ± 0.97	0.00
013	Linden leaf (*Tilia* L.)	Radzyń Podlaski (51°46′59″N, 22°37′00″E)	15.40 ± 0.71	206.67 ± 4.32	44.48 ± 3.02	45.75 ± 2.56	2.31 ± 0.10	7.47 ± 0.56	0.00
014	Linden leaf (*Tilia* L.)	Nakło nad Notecią (53°08′31″N, 17°36′06″E)	17.10 ± 0.28	212.90 ± 21.81	26.58 ± 0.95	58.06 ± 0.57	3.06 ± 0.02	11.01 ± 1.41	1.31 ± 0.14
015	Pine needles (*Pinus* L.)	Kalisz (51°45′39″N, 18°05′27″E)	16.05 ± 0.64	168.85 ± 7.62	43.29 ± 2.91	38.25 ± 0.99	3.59 ± 0.61	10.85 ± 0.87	1.90 ± 0.03
016	Air, street	Warszawa (52°13′44″N, 21°00′59″E)	14.05 ± 1.91	13.88 ± 1.26	0.00	0.00	19.45 ± 1.57	80.55 ± 1.57	00
017	Air, forest	Skarżysko-Kamienna (51°06′47″N, 20°52′17″E)	13.63 ± 0.33	76.77 ± 4.88	1.91 ± 0.78	22.24 ± 1.62	3.61 ± 1.19	6.43 ± 0.19	10.16 ± 0.47 19.30 ± 1.20 22.72 ± 0.64 13.66 ± 1.48
018	Air, garden	Piastów (52°11′03″N, 20°50′22″E)	13.67 ± 0.75	48.15 ± 0.79	8.71 ± 1.39	41.26 ± 1.87	19.85 ± 1.12	10.23 ± 0.30	19.97 ± 1.31
019	Air, forest	Skarżysko-Kamienna (51°06′47″N, 20°52′17″E)	13.15 ± 0.07	25.19 ± 0.81	0.00	70.35 ± 2.84	29.65 ± 2.84	0.00	0.00
020	Air, garden	Kraków (50°03′41″N, 19°56′11″E)	14.07 ± 0.28	228.55 ± 12.16	2.15 ± 0.25	53.19 ± 2.89	7.31 ± 0.01	37.36 ± 3.16	0.00
021	Air, garden	Nakło nad Notecią (53°08′31″N, 17°36′06″E)	9.03 ± 0.61	161.64 ± 19.45	33.38 ± 1.63	41.18 ± 1.34	4.94 ± 0.99	20.50 ± 1.27	0.00
022	Air, forest	Nadarzyn (52°05′39″N, 20°48′27″E)	4.93 ± 1.13	180.50 ± 8.19	14.67 ± 1.51	62.21 ± 1.76	1.29 ± 0.08	20.24 ± 3.72	1.59 ± 0.37
023	Air, street	Warszawa (52°13′44″N, 21°00′59″E)	17.65 ± 3.61	134.01 ± 22.37	15.84 ± 0.14	78.49 ± 0.08	0.00	5.68 ± 0.06	0.00
024	Air, garden	Pruszków (52°10′14″N, 20°48′43″E)	2.10 ± 0.33	190.18 ± 3.79	13.44 ± 1.54	30.45 ± 1.28	4.44 ± 1.28	49.74 ± 4.95	1.94 ± 0.85
025	Air, Botanic Garden	Warszawa (52°13′44″N, 21°00′59″E)	6.55 ± 1.06	142.46 ± 27.12	26.23 ± 3.48	46.66 ± 2.59	9.08 ± 0.16	18.03 ± 1.05	0.00
026	Air, Botanic Garden	Warszawa (52°13′44″N, 21°00′59″E)	14.77 ± 0.14	158.35 ± 12.29	65.49 ± 2.78	21.68 ± 0.14	2.88 ± 0.62	7.36 ± 1.72	2.60 ± 0.30
027	Air, street	Wrocław (51º6′32″N, 17º1′57″E)	11.80 ± 0.42	142.75 ± 14.34	26.79 ± 2.19	34.40 ± 2.90	7.00 ± 0.80	31.82 ± 1.51	0.00
028	Air, street	Warszawa (52°13′44″N, 21°00′59″E)	16.17 ± 0.42	200.23 ± 21.72	36.87 ± 1.23	45.45 ± 1.17	4.31 ± 0.19	12.03 ± 0.40	1.35 ± 0.26
029	Air, Botanic Garden	Warszawa (52°13′44″N, 21°00′59″E)	5.60 ± 0.42	115.59 ± 16.65	36.87 ± 1.23	45.45 ± 1.17	4.31 ± 0.19	12.03 ± 0.40	0.00
030	Air, garden	Piastów (52°11′03″N, 20°50′22″E)	13.37 ± 1.56	209.78 ± 21.33	30.87 ± 2.02	52.07 ± 1.89	4.29 ± 0.95	11.02 ± 0.86	1.77 ± 0.23
031	Air, Arboretum	Rogów (51°49′03″N, 19°53′11″E)	16.76 ± 0.04	284.31 ± 13.31	53.99 ± 0.52	32.38 ± 0.03	2.77 ± 0.09	8.64 ± 0.06	1.45 ± 0.18
032	Air, garden	Żyrardów (52°02′55″N, 20°26′45″E)	14.90 ± 1.27	14.24 ± 5.96	0.00	33.85 ± 1.97	3.85 ± 0.57	62.31 ± 2.54	0.00
033	Air, street	Kalisz (51°45′39″N, 18°05′27″E)	9.23 ± 0.90	197.34 ± 1.66	44.51 ± 5.24	11.65 ± 1.00	2.44 ± 0.45	41.41 ± 4.70	0.00
034	Air, forest	Nadarzyn (52°05′39″N, 20°48′27″E)	8.05 ± 0.92	124.41 ± 3.23	29.82 ± 2.53	39.57 ± 0.83	8.20 ± 0.69	22.42 ± 1.10	0.00
035	Air, street	Sokołów Podlaski (52°24′24″N, 22°15′11″E)	13.57 ± 0.33	179.87 ± 16.97	2.14 ± 0.45	38.88 ± 2.22	3.75 ± 0.70	55.24 ± 2.47	0.00
036	Air, university campus	Warszawa (52°13′44″N, 21°00′59″E)	12.18 ± 0.83	273.55 ± 12.93	0.00	42.39 ± 3.37	2.01 ± 0.17	55.60 ± 3.56	0.00
037	Air, street	Warszawa (52°13′44″N, 21°00′59″E)	9.15 ± 29.67	232.15 ± 29.67	14.56 ± 2.69	12.87 ± 1.55	6.29 ± 1.34	66.29 ± 2.90	0.00
038	Air, Botanic Garden	Warszawa (52°13′44″N, 21°00′59″E)	13.45 ± 1.34	248.29 ± 12.32	32.00 ± 3.47	39.85 ± 2.11	3.69 ± 1.01	24.47 ± 0.35	0.00
039	Air, university campus	Warszawa (52°13′44″N, 21°00′59″E)	7.35 ± 0.35	140.56 ± 10.37	9.84 ± 1.94	42.95 ± 3.38	3.12 ± 0.52	39.29 ± 1.12	4.81
040	Air, street	Koszalin (54°11′39″N, 16°10′19″E)	2.30 ± 0.42	131.25 ± 8.84	9.84 ± 1.97	42.95 ± 3.38	3.12 ± 0.52	39.29 ± 1.12	4.81 ± 0.81
041	Air, garden	Podkowa Leśna (52°07′20″N, 20°43′35″E)	12.50 ± 0.85	295.20 ± 5.90	73.67 ± 2.38	18.66 ± 2.05	1.38 ± 0.38	6.30 ± 0.71	0.00
042	Air, street	Pruszków (52°10′14″N, 20°48′43″E)	4.50 ± 0.42	37.20 ± 6.31	0.00	12.37 ± 0.07	13.84 ± 2.12	73.79 ± 2.05	0.00
043	Air, street	Pruszków (52°10′14″N, 20°48′43″E)	7.30 ± 0.28	234.21 ± 24.82	40.11 ± 0.85	26.01 ± 1.55	7.94 ± 1.41	25.95 ± 0.71	0.00
044	Air, street	Sokołów Podlaski (52°24′24″N, 22°15′11″E)	8.50 ± 0.71	235.59 ± 10.56	19.81 ± 2.02	31.24 ± 1.56	1.99 ± 0.34	46.97 ± 0.79	0.00
045	Air, university campus	Warszawa (52°13′44″N, 21°00′59″E)	12.00 ± 1.41	162.19 ± 21.32	19.81 ± 2.02	31.24 ± 1.56	1.99 ± 0.34	46.97 ± 0.79	0.00
046	Air, university campus	Warszawa (52°13′44″N, 21°00′59″E)	17.83 ± 1.46	255.79 ± 4.44	0.00	9.40 ± 1.23	1.76 ± 0.12	14.05 ± 1.69	74.80 ± 0.58
047	Air, Botanic Garden	Warszawa (52°13′44″N, 21°00′59″E)	10.15 ± 0.64	219.32 ± 3.30	9.07 ± 1.10	18.35 ± 1.43	5.57 ± 1.92	67.01 ± 0.61	0.00
048	Air, Botanic Garden	Warszawa (52°13′44″N, 21°00′59″E)	9.80 ± 0.71	268.12 ± 10.42	25.95 ± 2.26	60.01 ± 1.77	1.36 ± 0.26	12.69 ± 0.75	0.00
049	Air, street	Olsztyn (53º46′36″N, 20º28′40″E)	18.45 ± 0.21	145.76 ± 16.05	20.07 ± 3.97	53.01 ± 2.61	1.85 ± 0.01	25.09 ± 1.35	0.00
050	Air, street	Warszawa (52°13′44″N, 21°00′59″E)	14.75 ± 1.20	107.20 ± 10.44	11.03 ± 0.13	63.73 ± 2.18	1.95 ± 0.33	23.30 ± 1.98	0.00
051	Air, street	Warszawa (52°13′44″N, 21°00′59″E)	2.00 ± 0.85	224.59 ± 1.94	11.67 ± 1.16	22.04 ± 1.70	1.90 ± 0.62	41.30 ± 2.60	9.06 ± 1.97 14.04 ± 4.12
052	Air, street	Warszawa (52°13′44″N, 21°00′59″E)	10.55 ± 0.35	118.93 ± 8.58	17.57 ± 1.88	42.42 ± 2.01	5.15 ± 0.43	30.97 ± 0.06	3.90 ± 0.62
053	Redcurrant fruit (*Ribes spicatum*)	Skarżysko-Kamienna (51°06′47″N, 20°52′17″E)	19.30 ± 0.57	144.30 ± 12.93	24.45 ± 1.77	64.77 ± 2.27	0.43 ± 0.10	10.36 ± 0.60	0.00
054	Redcurrant fruit (*Ribes spicatum*)	Skarżysko-Kamienna (51°06′47″N, 20°52′17″E)	15.30 ± 0.57	206.67 ± 1.60	62.32 ± 4.35	15.64 ± 1.24	3.30 ± 1.18	17.18 ± 1.61	1.58 ± 0.33
055	Blackcurrant fruit (*Ribes nigrum*)	Gdynia (54°31′08″N, 18°31′54″E)	17.65 ± 0.35	155.08 ± 1.90	71.05 ± 3.58	24.35 ± 3.05	1.19 ± 0.21	3.42 ± 0.32	0.00
056	Wild strawberry fruit (*Fragaria* spp.)	Piastów (52°11′03″N, 20°50′22″E)	14.47 ± 0.38	234.72 ± 23.21	42.01 ± 2.45	22.37 ± 1.59	1.58 ± 0.34	27.95 ± 1.44	6.10 ± 0.92
057	Apple fruit (*Malus* spp.)	Piastów (52°11′03″N, 20°50′22″E)	17.65 ± 0.21	202.28 ± 13.95	34.72 ± 2.97	52.95 ± 2.74	0.48 ± 0.08	11.86 ± 0.30	0.00
058	Apple fruit (*Malus* spp.)	Gdynia (54°31′08″N, 18°31′54″E)	14.30 ± 0.00	338.72 ± 10.51	24.19 ± 1.66	41.90 ± 0.81	4.03 ± 0.21	29.89 ± 0.64	0.00
059	Kamchatka berry fruit (*Lonicera caerulea* var. *kamtschatica*)	Skarżysko-Kamienna (51°06′47″N, 20°52′17″E)	17.90 ± 1.84	134.24 ± 16.75	18.70 ± 1.27	70.34 ± 0.88	1.34 ± 0.08	9.63 ± 0.47	0.00
060	Strawberry fruit (*Fragaria* × *ananassa* Duchesne)	Gorzów Wielkopolski (52°44′12″N, 15°13′43″E)	18.70 ± 0.14	120.98 ± 1.92	63.27 ± 0.21	27.49 ± 0.78	0.44 ± 0.13	5.38 ± 0.86	0.00
061	Blueberry fruit (*Vaccinium corymbosum*)	Ostrowiec Świętokrzyski (50°51′N, 21°23′E)	16.25 ± 1.48	209.15 ± 6.60	27.97 ± 2.66	39.67 ± 1.02	0.57 ± 0.13	31.79 ± 1.51	0.00
062	Pumpkin fruit (*Cucurbita pepo* L.)	Piastów (52°11′03″N, 20°50′22″E)	16.70 ± 0.71	198.74 ± 0.48	41.30 ± 0.49	42.33 ± 0.93	2.36 ± 0.08	14.02 ± 0.36	0.00
063	Grape (*Vitis vinifera* L.)	Skarżysko-Kamienna (51°06′47″N, 20°52′17″E)	17.75 ± 0.64	185.64 ± 4.17	56.00 ± 3.28	38.85 ± 3.71	2.05 ± 0.30	3.11 ± 0.13	0.00
064	Grape (*Vitis vinifera* L.)	Skarżysko-Kamienna (51°06′47″N, 20°52′17″E)	16.30 ± 0.42	269.62 ± 10.92	29.36 ± 1.49	43.92 ± 2.04	0.64 ± 0.16	14.95 ± 0.09	7.22 ± 0.06 3.93 ± 0.68
065	Homemade fermented carrot juice	Warszawa (52°13′44″N, 21°00′59″E)	16.60 ± 1.27	110.14 ± 5.25	2.08 ± 0.18	91.39 ± 1.85	1.65 ± 0.47	4.89 ± 1.21	0.00
066	Tomato fruit (*Solanum lycopersicum*)	Kraków (50°03′41″N, 19°56′11″E)	13.55 ± 0.92	94.05 ± 12.25	29.79 ± 1.48	48.06 ± 3.15	1.83 ± 0.14	20.33 ± 4.48	0.00
067	Rose petals (*Rosa* L.)	Kraków (50°03′41″N, 19°56′11″E)	17.50 ± 1.56	184.86 ± 20.98	17.12 ± 0.28	66.25 ± 1.49	1.14 ± 0.15	15.51 ± 1.07	0.00
068	Rose petals (*Rosa* L.)	Tomaszów Mazowiecki (51°31′52″N, 20°00′30″E)	16.53 ± 1.04	231.24 ± 20.56	33.48 ± 0.75	56.07 ± 2.64	2.73 ± 0.02	7.73 ± 1.92	0.00
069	Rose petals (*Rosa* L.)	Żyrardów (52°02′55″N, 20°26′45″E)	16.20 ± 0.28	116.48 ± 3.42	13.71 ± 0.66	78.37 ± 1.43	0.66 ± 0.18	7.27 ± 0.59	0.00
070	Lily flower (*Lilium* L.)	Koszalin (54°11′39″N, 16°10′19″E)	14.65 ± 0.07	212.43 ± 12.25	21.07 ± 0.33	37.66 ± 0.33	4.32 ± 0.09	36.96 ± 0.09	0.00
071	Lily flower (*Lilium* L.)	Warka (51°47′03″N, 21°11′27″E)	17.90 ± 0.71	156.06 ± 17.03	18.89 ± 0.21	70.43 ± 0.16	1.69 ± 0.02	9.00 ± 0.32	0.00
072	Rhododendron flowers (*Rhododendron* L.)	Rogów (51°49′03″N, 19°53′11)	16.10 ± 0.14	172.06 ± 20.73	13.35 ± 0.06	60.12 ± 0.06	9.53 ± 1.54	17.01 ± 1.55	0.00
073	Rhododendron flowers (*Rhododendron* L.)	Rogów (51°49′03″N, 19°53′11)	14.75 ± 1.48	178.57 ± 28.76	30.97 ± 0.07	47.28 ± 1.05	0.67 ± 0.18	17.47 ± 1.70	3.62 ± 0.76
074	Rhododendron flowers (*Rhododendron* L.)	Rogów (51°49′03″N, 19°53′11)	16.60 ± 1.13	158.39 ± 7.60	13.60 ± 2.61	50.63 ± 1.32	9.82 ± 0.07	25.96 ± 1.36	0.00
075	Rhododendron flowers (*Rhododendron* L.)	Warszawa (52°13′44″N, 21°00′59″E)	15.65 ± 0.35	194.61 ± 10.61	26.46 ± 2.03	52.20 ± 1.62	2.91 ± 0.11	18.44 ± 0.52	0.00
076	Rhododendron flowers (*Rhododendron* L.)	Tomaszów Mazowiecki (51°31′52″N, 20°00′30″E)	14.25 ± 0.07	292.55 ± 2.07	9.97 ± 0.08	55.68 ± 0.35	2.00 ± 0.03	31.62 ± 0.27	0.74 ± 0.13
077	Tulip flowers (*Tulipa* L.)	Sokołów Podlaski (52°24′24″N, 22°15′11″E)	13.65 ± 1.91	217.89 ± 25.29	9.90 ± 1.80	64.40 ± 0.83	5.07 ± 0.49	20.64 ± 0.48	0.00
078	Tulip flowers (*Tulipa* L.)	Piastów (52°11′03″N, 20°50′22″E)	16.95 ± 0.07	193.22 ± 3.93	38.54 ± 4.79	38.12 ± 2.46	2.90 ± 0.36	16.51 ± 1.37	3.94 ± 1.32
079	Marigold flowers (*Tagetes* L.)	Warszawa (52°13′44″N, 21°00′59″E)	6.10 ± 0.28	204.35 ± 19.62	31.94 ± 0.24	50.59 ± 0.54	2.82 ± 0.42	13.31 ± 0.18	1.34 ± 0.18
080	Marigold flowers (*Tagetes* L.)	Warszawa (52°13′44″N, 21°00′59″E)	15.15 ± 0.49	198.10 ± 30.39	31.94 ± 0.24	50.59 ± 0.54	2.82 ± 0.42	13.31 ± 0.18	0.00
081	Marigold flowers (*Tagetes* L.)	Gorzów Wielkopolski (52°44′12″N, 15°13′43″E)	15.30 ± 1.70	176.60 ± 9.77	11.29 ± 1.34	62.70 ± 0.64	8.92 ± 0.76	17.11 ± 0.06	0.00
082	Marigold flowers (*Tagetes* L.)	Warszawa (52°13′44″N, 21°00′59″E)	9.20 ± 0.99	120.57 ± 18.74	12.93 ± 1.41	63.61 ± 0.45	2.02 ± 0.14	21.45 ± 1.10	0.00
083	Cornflower flowers (*Centaurea cyanus*)	Opole (50°40′19″N, 17°55′31″E)	11.15 ± 0.07	49.32 ± 2.86	23.24 ± 3.03	48.15 ± 2.96	2.59 ± 0.19	26.03 ± 0.11	0.00
084	Cornflower flowers (*Centaurea cyanus*)	Tomaszów Mazowiecki (51°31′52″N, 20°00′30″E)	16.00 ± 2.64	70.90 ± 19.80	4.35 ± 1.75	63.69 ± 1.14	2.04 ± 0.42	29.93 ± 1.04	0.00
085	Cornflower flowers (*Centaurea cyanus*)	Kołobrzeg (54°10′32″N, 15°35′00″E)	10.80 ± 0.42	176.62 ± 6.12	50.57 ± 3.34	39.68 ± 2.22	1.60 ± 0.33	8.16 ± 1.45	0.00
086	Cornflower flowers (*Centaurea cyanus*)	Kołobrzeg (54°10′32″N, 15°35′00″E)	8.90 ± 0.42	231.91 ± 7.08	70.12 ± 3.23	20.59 ± 1.90	0.79 ± 0.08	8.52 ± 1.41	0.00
087	Common poppy flowers (*Papaver rhoeas*)	Opole (50°40′19″N, 17°55′31″E)	17.30 ± 0.85	270.13 ± 10.69	17.87 ± 3.83	52.48 ± 1.71	3.33 ± 0.39	26.33 ± 1.73	0.00
088	Rudbeckia flowers (*Rudbeckia* L.)	Piastów (52°11′03″N, 20°50′22″E)	15.60 ± 1.70	224.70 ± 13.68	41.44 ± 0.47	48.65 ± 2.51	2.67 ± 0.81	6.23 ± 1.26	1.02 ± 0.03
089	Rudbeckia flowers (*Rudbeckia* L.)	Nadarzyn (52°05′39″N, 20°48′27″E)	15.87 ± 0.09	127.58 ± 16.33	5.77 ± 0.61	59.71 ± 2.54	3.95 ± 0.57	30.58 ± 1.36	0.00
090	Rudbeckia flowers (*Rudbeckia* L.)	Sokołów Podlaski (52°24′24″N, 22°15′11″E))	3.65 ± 0.35	158.51 ± 20.20	44.83 ± 2.19	28.51 ± 1.49	5.79 ± 0.78	20.88 ± 2.91	0.00
091	Rudbeckia flowers (*Rudbeckia* L.)	Sokołów Podlaski (52°24′24″N, 22°15′11″E)	13.47 ± 0.57	161.28 ± 10.73	10.79 ± 1.56	48.63 ± 0.70	6.64 ± 1.29	33.95 ± 0.98	0.00
092	Hydrangea flowers (*Hydrangea* L.)	Warszawa (52°13′44″N, 21°00′59″E)	18.57 ± 1.18	109.98 ± 0.63	14.62 ± 1.46	54.06 ± 0.79	8.15 ± 1.36	23.17 ± 0.89	0.00
093	Hydrangea flowers (*Hydrangea* L.)	Żyrardów (52°02′55″N, 20°26′45″E)	19.55 ± 1.91	168.95 ± 9.72	20.17 ± 0.11	66.02 ± 0.11	2.05 ± 0.12	11.77 ± 1.32	0.00
094	Flowers of the delphinium (*Delphinium consolida* L.)	Sokołów Podlaski (52°24′24″N, 22°15′11″E)	14.43 ± 0.52	181.47 ± 22.06	12.94 ± 2.18	70.64 ± 2.48	3.86 ± 0.57	12.58 ± 0.28	0.00
095	Blue phacelia flowers (*Phacelia tanacetifolia*)	Sokołów Podlaski (52°24′24″N, 22°15′11″E)	16.60 ± 0.57	168.55 ± 14.80	65.74 ± 0.34	19.68 ± 0.45	3.41 ± 0.23	9.55 ± 0.22	1.64 ± 0.11
096	Common chamomile flower (*Matricaria chamomilla* L.)	Nadarzyn (52°05′39″N, 20°48′27″E)	15.00 ± 0.57	161.51 ± 13.75	49.39 ± 2.69	33.23 ± 1.36	4.77 ± 0.26	10.45 ± 1.52	2.18 ± 0.45
097	Alfalfa flower (*Medicago sativa* L.)	Radzyń Podlaski (51°46′59″N, 22°37′00″E)	7.25 ± 0.49	103.36 ± 2.70	30.20 ± 2.84	37.80 ± 2.04	8.84 ± 0.40	23.16 ± 0.41	0.00
098	*Thymus alpestris* flowers	Zakopane (49°17′58″N, 19°57′07″E)	12.57 ± 2.03	68.90 ± 7.36	3.50 ± 0.16	44.54 ± 0.37	17.68 ± 0.01	34.29 ± 0.54	0.00
099	Goldenrod flowers (*Solidago virgaurea* L.)	Ryki (51°37.5444′N, 21°55.9644′E)	17.30 ± 0.99	214.40 ± 5.63	64.06 ± 0.21	22.19 ± 0.48	3.60 ± 0.04	6.39 ± 0.88	3.77 ± 0.16
100	*Sonchus arvensis* L. flowers	Ryki (51°37.5444′N, 21°55.9644′E)	15.85 ± 0.49	216.68 ± 12.90	43.31 ± 1.56	41.52 ± 2.98	4.05 ± 1.40	9.94 ± 0.09	1.20 ± 0.11
101	Flowers hypochaeris rough (*Hypochaeris radicata* L.)	Ustrzyki górne (49°06′17″N 22°39′02″E)	17.07 ± 1.23	214.09 ± 7.41	38.29 ± 3.03	48.06 ± 2.89	1.83 ± 0.09	11.83 ± 0.23	0.00
102	*Cosmos bipinnatus* flowers	Warszawa (52°13′44″N, 21°00′59″E)	14.20 ± 0.42	226.46 ± 15.48	59.29 ± 0.89	32.02 ± 0.23	1.68 ± 0.02	7.02 ± 0.64	0.00
103	A blade of *Avena* L.	Rogów (51°49′03″N, 19°53′11)	13.63 ± 1.56	143.67 ± 11.21	67.81 ± 0.25	25.06 ± 1.00	3.35 ± 0.81	2.15 ± 0.06	1.64 ± 0.11
104	A blade of *Avena* L.	Rogów (51°49′03″N, 19°53′11)	14.30 ± 1.56	243.69 ± 7.14	19.97 ± 1.23	43.50 ± 0.29	2.16 ± 0.30	34.38 ± 0.64	0.00
105	A blade of *Avena* L.	Warszawa (52°13′44″N, 21°00′59″E)	10.05 ± 1.06	108.61 ± 19.38	8.30 ± 0.08	71.46 ± 2.52	2.09 ± 0.36	18.17 ± 2,81	0.00
106	Feta cheese	Warszawa (52°13′44″N, 21°00′59″E)	14.03 ± 0.42	194.28 ± 6.71	81.97 ± 2.26	2.73 ± 0.57	2.95 ± 1.09	9.32 ± 1.46	3.04 ± 1.32
107	Minced pork	Warszawa (52°13′44″N, 21°00′59″E)	18.05 ± 0.64	163.14 ± 16.77	18.29 ± 0.71	67.56 ± 1.39	2.30 ± 0.22	11.86 ± 1.87	0.00
108	Bee pollen	Białystok (53°08′07″N, 23°08′44″E)	17.80 ± 1.70	157.41 ± 9.55	9.69 ± 0.49	77.02 ± 1.54	2.20 ± 0.62	10.60 ± 1.42	0.00
109	Bee bread	Białystok (53°08′07″N, 23°08′44″E)	15.10 ± 0.57	137.63 ± 10.65	2.51 ± 0.47	86.48 ± 1.34	1.72 ± 0.02	9.30 ± 0.85	0.00
110	Homemade yogurt	Warszawa (52°13′44″N, 21°00′59″E)	14.39 ± 0.85	221.92 ± 13.04	34.13 ± 2.46	52.35 ± 4.45	1.83 ± 0.14	11.69 ± 0.73	0.00
111	Salda mix	Warszawa (52°13′44″N, 21°00′59″E)	16.95 ± 0.07	232.47 ± 18.05	20.66 ± 3.31	48.98 ± 1.99	5.16 ± 0.45	25.21 ± 0.87	0.00
112	Chocolate bar	Warszawa (52°13′44″N, 21°00′59″E)	15.70 ± 2.55	166.42 ± 10.09	18.80 ± 2.38	52.89 ± 2.43	4.44 ± 1.36	23.88 ± 1.32	0.00
113	Laboratory contamination	Warszawa (52°13′44″N, 21°00′59″E)	16.20 ± 2.07	168.52 ± 8.85	12.32 ± 1.34	55.68 ± 2.96	2.26 ± 0.12	29.75 ± 4.43	0.00
114	Sour cream	Radom (51°24′09″N, 21°08′49″E	11.33 ± 0.28	406.50 ± 9.61	50.30 ± 3.35	41.05 ± 2.28	1.74 ± 0.39	6.92 ± 0.69	0.00

The isolated yeast strains produced carotenoid pigments, the total content of which in the cellular biomass ranged from 13.88 to 406.50 µg/g (Table 1). In the available literature, different values are provided for the content of carotenoids synthesized by microorganisms. In order to sort them out, El-Banna et al. [15] proposed the classification of carotenoid-producing strains into low-efficiency (below 100 µg/g), medium-efficiency (between 100 and 500 µg/g), and high-efficiency (above 500 µg/g) strains. Among the investigated red yeast strains, 10 can be considered as low-efficiency producers, and the remaining 104 strains as medium-efficiency producers, of which 61 isolates synthesized carotenoids at an amount of 101.84–198.74 µg/g and 41 strains in the range of 200.23–295.20 µg/g. The other two strains synthesized significantly more carotenoids. The greatest amount of total carotenoids was produced by the A-058 strain (338.72 µg/g) isolated from apple and the A-114 strain (406.50 µg/g) which was originally a contaminant of cream stored under refrigerated conditions.

The carotenoid-biosynthesizing capacity of yeasts depends primarily on the strain and culture conditions. Of the 16 red yeast strains isolated by Chreptowicz et al. [14], *Sporobolomyces roseus* WUT182 synthesized as much as 1.12 mg of carotenoids per gram of cellular biomass. In another work by Allahkarami et al. [16], red yeast was isolated from Pinaceae forest ecosystems. The most efficient of the isolated strains, *R. mucilaginosa*, synthesized only 223.5 µg/g. In the study by Libkind and Van Broock [17], 15 strains of carotenogenic yeast were isolated from the natural environment in northwestern Patagonia. Similar to the work of Allahkarami et al. [16] the highest amount of carotenoids (301 µg/g_d.m_.) was synthesized by a *R. mucilaginosa* strain. The strains with the highest biomass yield were characterized by lower overall content of carotenoids in the cellular biomass.

The next stage of the present study was to determine the percentages of torularhodin, torulene, β-carotene, and γ-carotene. Torulene occurs in the highest amounts in yeast cells [18], which is confirmed by the results of this study. Among the isolated yeast strains, torulene represented more than 50% of the total carotenoid pool in 49 strains. Only in two strains (A-016 and A-007), this compound was not detected, while four strains (A-065, A-003, A-109, and A-005) had more than 80% of torulene in the total carotenoid pool (Table 1).

Currently, there is a demand for yeasts that can synthesize high amounts of torularhodin due to the anticancer properties of this compound [19]. In this study, seven isolated strains (A-016, A-042, A-032, A-036, A-007, A-046, and A-019) were not found to synthesize this carotenoid. Only in the case of 15 strains, torularhodin constituted more than 50% of the total carotenoid pool (Table 1). Strain A-106, which was originally isolated as a contaminant in feta cheese stored under refrigerated conditions, produced the highest amount of this compound (81.97%).

Eight yeast strains (A-016, A-042, A-047, A-037, A-032, A-040, A-035, and A-036) synthesized more than 50% of β-carotene in the carotenoid pool. β-Carotene was not detected in the biomass of only one yeast isolate (A-019). Interestingly, this strain synthesized the highest amount of γ-carotene (29.65%) which was found in the smallest amount in most of the isolated strains.

**Fig. 2:**
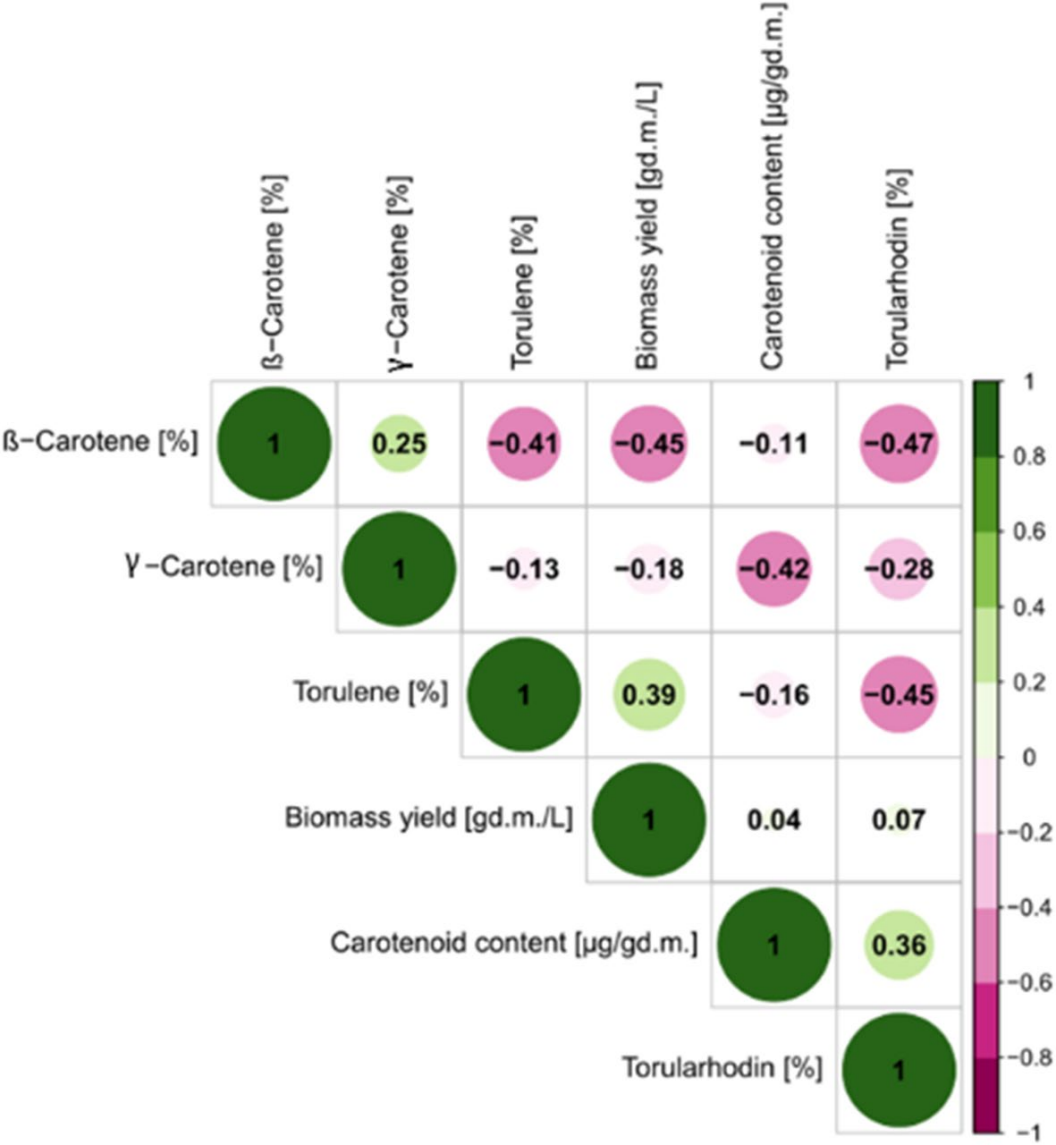
Correlations between the yield of cellular biomass, total carotenoid content and the shares of individual carotenoid fractions (β-carotene, γ-carotene, torulene and torularhodin)

A positive correlation was found between the content of β-carotene and γ-carotene. This can be attributed to the biosynthetic pathway of these compounds. γ-Carotene is a direct precursor of β-carotene [3]. The strains that synthesized more β-carotene and γ-carotene produced a less amount of cellular biomass. The opposite tendency was observed in the case of torulene: a positive correlation was found between the yield of cellular biomass and the content of this compound (Fig. 2). A positive correlation was also observed between the total content of carotenoids and the share of torularhodin.

### Identification of Selected Yeast Strains

To identify the genus and species of the yeast strains, a selection process was carried out. Yeasts were identified both genotypically and phenotypically. Strains that synthesized more than 200 µg of carotenoids per gram of dry matter (A-002, A-009, A-010, A-012, A-013, A-014, A-020, A-028, A-030, A-031, A-036, A-037, A-038, A-041, A-043, A-044, A-046, A-047, A-04, A-051, A-054, A-056, A-057, A-058, A-061, A-064, A-068, A-070, A-076, A-077, A-079, A-086, A-087, A-088, A-099, A-100, A-101, A-102, A-104, A-110, A-111 and A-114) were selected for further analysis. Those strains that synthesized more than 60% torularhodin (A-026, A-041, A-054, A-055, A-060, A-095, A-099, A-103 and A-106) were also selected. A total of 48 strains of yeast have been identified. The identification process revealed that the selected isolates belonged to the following genera: *Rhodotorula*, *Sporidiobolus*, *Sporobolomyces*, *Buckleyzyma*, *Cystofilobasidium*, and *Erythrobasidium* (Table 2).

**Table 2:** Red yeast strains identified by sequence comparison with the BLASTn match with the NCBI GenBank database

No.	Strain no.	Top BLAST search results	Query cover (%)	Identity (%)	Proposed taxa (GenBank acc. number)	CMIFS collection number
1	A-002	*Rhodotorula mucilaginosa*	100	100	*Rhodotorula mucilaginosa* (OM256524)	CMIFS 004
2	A-009	*Rhodotorula graminis*	100	100	*Rhodotorula babjevae* (ON202973)	CMIFS 020
		*Rhodotorula babjevae*	100	100		
		*Rhodotorula glutinis*	100	100		
3	A-010	*Rhodotorula graminis*	100	100	*Rhodotorula babjevae* (OQ996825)	CMIFS 094
		*Rhodotorula babjevae*	100	100		
		*Rhodotorula glutinis*	100	100		
		*Rhodotorula diobovata*	100	100		
4	A-012	*Sporidiobolus pararoseus*	100	100	*Sporidiobolus pararoseus* (ON202961)	CMIFS 012
		*Sporidiobolus* sp.	100	100		
		*Rhodotorula* sp.	100	100		
5	A-013	*Rhodotorula babjevae*	99	100	*Rhodotorula babjevae* (OQ996826)	CMIFS 096
		*Rhodotorula glutinis*	99	100		
		*Rhodotorula diobovata*	99	100		
		*Rhodotorula graminis*	99	100		
6	A-014	*Rhodotorula babjevae*	100	100	*Rhodotorula babjevae* (OQ990346)	CMIFS 040
		*Rhodotorula glutinis*	99	99.82		
		*Rhodotorula diobovata*	99	99.82		
7	A-020	*Rhodotorula graminis*	100	100	*Rhodotorula graminis* (ON203044)	CMIFS 030
		*Rhodotorula babjevae*	100	100		
		*Rhodotorula glutinis*	100	100		
8	A-026	*Rhodotorula graminis*	100	99.81	*Rhodotorula babjevae* (ON202975)	CMIFS 021
		*Rhodotorula babjevae*	100	99.81		
		*Rhodotorula glutinis*	100	99.81		
		*Rhodotorula diobovata*	100	99.81		
		*Ustilentyloma graminis*	100	99.81		
9	A-028	*Rhodotorula mucilaginosa*	100	100	*Rhodotorula mucilaginosa* (OR100603)	CMIFS 167
		*Rhodotorula alborubescens*	100	100		
10	A-030	*Rhodotorula graminis*	100	100	*Rhodotorula babjevae* (OR020690)	CMIFS 123
		*Rhodotorula babjevae*	100	100		
		*Rhodotorula glutinis*	100	100		
		*Ustilentyloma graminis*	100	100		
		*Rhodotorula diobovata*	100	100		
11	A-031	*Rhodotorula diobovata*	100	100	*Rhodotorula glutinis* (ON203045)	CMIFS 032
		*Rhodotorula glutinis*	100	100		
		*Rhodotorula graminis*	99	100		
12	A-036	*Rhodotorula diobovata*	100	99.81	*Rhodotorula babjevae* (OR032918)	CMIFS 171
		*Rhodotorula glutinis*	100	99.81		
		*Rhodotorula graminis*	100	99.81		
		*Rhodotorula babjevae*	100	99.81		
13	A-037	*Rhodotorula diobovata*	100	100	*Rhodotorula graminis* (OQ996256)	CMIFS 031
		*Rhodotorula glutinis*	100	100		
		*Rhodotorula graminis*	100	100		
		*Rhodotorula babjevae*	100	100		
14	A-038	*Sporidiobolus pararoseus*	100	100	*Sporidiobolus pararoseus* (ON202963)	CMIFS 013
		*Sporidiobolus* sp.	100	100		
		*Sporobolomyces* sp.	100	100		
15	A-041	*Rhodotorula babjevae*	100	100	*Rhodotorula babjevae* (OQ826838)	CMIFS 022
		*Rhodotorula diobovata*	100	100		
		*Rhodotorula glutinis*	100	100		
16	A-043	*Sporobolomyces ruberrimus*	100	100	*Sporobolomyces ruberrimus* (ON202960)	CMIFS 010
		*Sporidiobolus metaroseus*	100	100		
17	A-044	*Sporidiobolus metaroseus*	100	100	*Sporobolomyces roseus* (ON202962)	CMIFS 011
		*Sporobolomyces roseus*	100	100		
		*Sporobolomyces* sp.	100	100		
18	A-046	*Buckleyzyma aurantiaca*	100	99.82	*Buckleyzyma aurantiaca* (OM305030)	CMIFS 009
		*Rhodotorula* sp.	100	99.82		
19	A-047	*Rhodotorula mucilaginosa*	100	100	*Rhodotorula mucilaginosa* (OR100624)	CMIFS 170
20	A-048	*Rhodotorula diobovata*	100	100	*Rhodotorula diobovata* (OQ826837)	CMIFS 019
21	A-051	*Erythrobasidium hasegawianum*	100	100	*Erythrobasidium hasegawianum* (ON202967)	CMIFS 018
22	A-054	*Rhodotorula graminis*	100	100	*Rhodotorula babjevae* (OQ828067)	CMIFS 023
		*Rhodotorula babjevae*	100	100		
		*Rhodotorula glutinis*	100	100		
		*Rhodotorula diobovata*	100	100		
		*Ustilentyloma graminis*	100	100		
23	A-055	*Rhodotorula glutinis*	100	100	*Rhodotorula glutinis* (OQ828070)	CMIFS 033
		*Rhodotorula diobovata*	100	100		
24	A-056	*Cystofilobasidium macerans*	100	100	*Cystofilobasidium macerans* (ON202966)	CMIFS 017
25	A-057	*Rhodotorula mucilaginosa*	100	100	*Rhodotorula mucilaginosa* (OR100518)	CMIFS 165
		*Rhodotorula alborubescens*	100	100		
26	A-058	*Sporidiobolus pararoseus*	100	100	*Sporidiobolus pararoseus* (ON202964)	CMIFS 014
		*Sporidiobolus* sp.	100	100		
		*Sporobolomyces* sp.	100	100		
27	A-060	*Rhodotorula mucilaginosa*	100	100	*Rhodotorula mucilaginosa* (OQ826830)	CMIFS 005
28	A-061	*Rhodotorula taiwanensis*	100	100	*Rhodotorula taiwanensis* (OR036248)	CMIFS 164
29	A-064	*Rhodotorula graminis*	100	100	*Rhodotorula* sp. (ON203116)	CMIFS 035
		*Rhodotorula babjevae*	100	100		
30	A-068	*Rhodotorula graminis*	100	100	*Rhodotorula babjevae* (ON340724)	CMIFS 024
		*Rhodotorula babjevae*	100	100		
		*Rhodotorula glutinis*	100	100		
		*Ustilentyloma graminis*	100	100		
31	A-070	*Rhodotorula graminis*	100	100	*Rhodotorula babjevae* (OR004714)	CMIFS 091
		*Rhodotorula babjevae*	100	100		
		*Rhodotorula glutinis*	100	100		
		*Rhodotorula diobovata*	100	100		
32	A-076	*Sporidiobolus pararoseus*	95	99.82	*Sporobolomyces* sp. (ON202965)	CMIFS 016
		*Sporobolomyces* sp.	95	99.82		
33	A-077	*Rhodotorula mucilaginosa*	100	100	*Rhodotorula mucilaginosa* (OR100595)	CMIFS 166
		*Rhodotorula alborubescens*	100	100		
34	A-079	*Cystofilobasidium infirmominiatum*	100	100	*Cystofilobasidium infirmominiatum* (OQ197480)	CMIFS 048
		*Cystofilobasidium* sp.	100	100		
35	A-086	*Rhodotorula graminis*	100	100	*Rhodotorula babjevae* (OQ828069)	CMIFS 025
		*Rhodotorula babjevae*	100	100		
		*Rhodotorula glutinis*	100	100		
		*Rhodotorula diobovata*	100	100		
		*Ustilentyloma graminis*	100	100		
36	A-087	*Rhodotorula toruloides*	100	100	*Rhodotorula toruloides* (OQ990341)	CMIFS 007
37	A-088	*Rhodotorula graminis*	100	100	*Rhodotorula glutinis* (ON204010)	CMIFS 034
		*Rhodotorula babjevae*	100	100		
		*Rhodotorula glutinis*	100	100		
		*Rhodotorula diobovata*	100	100		
		*Ustilentyloma graminis*	100	100		
38	A-095	*Rhodotorula graminis*	100	100	*Rhodotorula babjevae* (ON203025)	CMIFS 026
		*Rhodotorula babjevae*	100	100		
		*Rhodotorula glutinis*	100	100		
		*Rhodotorula diobovata*	100	100		
		*Ustilentyloma graminis*	100	100		
39	A-099	*Rhodotorula graminis*	100	100	*Rhodotorula babjevae* (ON203027)	CMIFS 027
		*Rhodotorula babjevae*	100	100		
		*Rhodotorula glutinis*	100	100		
		*Rhodotorula diobovata*	100	100		
40	A-100	*Rhodotorula graminis*	100	100	*Rhodotorula babjevae* (OR039570)	CMIFS 169
		*Rhodotorula babjevae*	100	100		
		*Rhodotorula glutinis*	100	100		
		*Rhodotorula diobovata*	100	100		
41	A-101	*Rhodotorula glutinis*	100	99.81	*Rhodotorula glutinis* (OR039629)	CMIFS 126
		*Rhodotorula babjevae*	100	99.81		
		*Rhodotorula diobovata*	100	99.81		
42	A-102	*Rhodotorula graminis*	96	100	*Rhodotorula babjevae* (ON203028)	CMIFS 028
		*Rhodotorula babjevae*	95	100		
43	A-103	*Rhodotorula graminis*	100	100	*Rhodotorula babjevae* (ON203029)	CMIFS 029
		*Rhodotorula babjevae*	100	100		
		*Rhodotorula glutinis*	100	100		
		*Rhodotorula diobovata*	100	100		
		*Ustilentyloma graminis*	100	100		
44	A-104	*Sporidiobolus pararoseus*	100	100	*Sporidiobolus pararoseus* (ON204008)	CMIFS 015
		*Sporidiobolus* sp.	100	100		
		*Rhodotorula* sp.	100	100		
45	A-106	*Rhodotorula graminis*	100	99.45	*Rhodotorula* sp. (ON203118)	CMIFS 036
		*Rhodotorula babjevae*	100	99.45		
		*Rhodotorula glutinis*	100	99.45		
		*Rhodotorula diobovata*	100	99.45		
		*Ustilentyloma graminis*	100	99.45		
46	A-110	*Rhodotorula babjevae*	99	100	*Rhodotorula babjevae* (OR041764)	CMIFS 163
		*Rhodotorula graminis*	99	100		
47	A-111	*Rhodotorula toruloides*	100	99.81	*Rhodotorula toruloides * (OM281704)	CMIFS 008
		*Rhodosporidium toruloides*	100	99.81		
48	A-114	*Rhodotorula mucilaginosa*	100	100	*Rhodotorula mucilaginosa* (OM257175)	CMIFS 006
		*Rhodotorula alborubescens*	100	100		

Seven red yeast strains (A-002, A-028, A-047, A-057, A-060, A-077 and A-114) were identified as *Rhodotorula mucilaginosa*. The sequences of the LSU domain of strains A-002, A-047 and A-060 showed 100% similarity only to *R. mucilaginosa*, while strains A-028, A-057, A-077 and A-114 also to *Rhodotorula alborubescens*. This species was introduced during a phylogenetic revision of the yeast subdivision Pucciniomycotina by Wang et al. in 2015 [20]. Its basionym is *Sporobolomyces alborubescens*, which was first described in 1930 by Derx [21]. *Rhodotorula mucilaginosa* and *Sporobolomyces alborubescens* have an identical sequence of the LSU domain and are physiologically very similar, while the characteristic feature of the yeast *S. alborubescens* is the ability to use D-glucosamine as a nitrogen source [22]. Furthermore, the yeast *R. alborubescens* CBS 482, recognized as a typical representative of this species, does not show the ability to assimilate sorbitol and citric acid, but assimilates L-tartaric acid and Tween 80 [23]. All tested isolates did not assimilate D-glucosamine (nitrogen source) and L-tartaric acid and Tween 80 (as carbon sources), but they were able to metabolize sorbitol and citric acid as carbon sources (Table 3). The species *R. mucilaginosa* is cosmopolitan and has been isolated from natural environments worldwide [8]. It has wide biotechnological applications. Depending on the strain, *R. mucilaginosa* exhibits the ability to biosynthesize exopolysaccharides [20], patulin-degrading aldolases [24], carotenoids [25], proteases [26], and Single Cell Oil (SCO) [27], and can be used as a biological agent in biocontrol [28]. The yeast *R. mucilaginosa* is also characterized by detoxification mechanisms that can neutralize the toxic effects of various elements (including selenium) present in the culture medium by forming selenium nanoparticles. Furthermore, this species can transform inorganic selenium into organic and elemental forms (Se0) [29].

**Table 3:** Physiological characteristics of *Rhodotorula mucilaginosa* and *Buckleyzyma aurantiaca* isolates

Assimilation	A-002	A-028	A-046	A-047	A-057	A-060	A-077	A-114	R. mucilaginosa**	B. aurantiaca*
Glucose	+	+	+	+	+	+	+	+	+	+
D-Xylose	+	+	+	+	+	+	+	+	+	+
Galactose	+	+	w	+	s	+	s	+	+/s	–/w/s
L-Arabinose	+	+	+	+	+	+	+	+	+	+
L-Rhamnose	–	–	–	–	–	–	–	–	–	–
Fructose	+	+	–	+	+	+	+	+	n	n
Maltose	+	+	s	+	+	+	+	+	+	s
Saccharose	+	+	+	+	+	+	+	+	+	+
Lactose	–	–	–	–	–	–	–	–	–	–
Glycerol	+	+	+	+	+	+	+	+	+	+
D-Mannitol	+	+	+	+	s	+	+	+	v	+
Trehalose	+	+	–	+	+	+	+	+	+	v
Xylitol	–	+	–	+	+	+	+	–	+	–/w/s
Erythritol	–	–	–	–	–	–	–	–	–	–
Inulin	–	–	–	–	–	–	–	–	–	–
DL-Lactate	+	+	–	+	+	+	+	+	+	–/s
L-Tartaric acid	–	–	–	–	–	–	–	–	–	–
Citrate	+	+	+	+	+	+	+	+	+	+
Ethanol	+	+	+	+	+	+	+	+	+	+/w/s
Methanol	–	–	–	–	–	–	–	–	–	–
Raffinose	+	+	–	+	+	+	+	+	+	–
Nitrates	–	–	–	–	–	–	–	–	–	v
D-glucosamine	–	–	x	–	–	–	–	–	–	–
Tween 80	–	–	x	–	–	–	–	–	–	n
Sorbitol	+	+	x	+	+	+	+	+	+	n
Vanillic acid	+	+	–	+	+	+	+	+	+	–
Gallic acid	–	–	–	–	–	–	–	–	–	–
Vitamins-free	–	–	–	–	–	–	–	–	–	–
Growth at 35 °C	+	+	–	-	+	–	+	+	v	–

*Typical strains from [8]; ** Typical strains from [8] and [22, 23]; +, positive; l, latent (rapid development of a positive reaction after a lag period); s, positive but slow; w, weak; ws, weak and slow; lw, latent but weak (rapid development of a weak reaction after a lag period); v, variable; –, negative; n, no data; x - not determined

The strain A-046 was identified as *Buckleyzyma aurantiaca* (Table 2). Previously, this species was classified as *Rhodotorula aurantiaca*. Later, in 2015, a new genus *Buckleyzyma* was created by Wang and colleagues, and the species *B. aurantiaca* was considered typical of this genus. In this study, the A-046 isolate grew slowly on the YPD agar and formed small, intense orange colonies. In assimilation tests, it showed significant similarity to the strains described in “The Yeasts—A Taxonomic Study” (Table 3). A characteristic feature of the isolate was that it could not grow at temperatures above 25℃. Some strains of *B. aurantiaca* have been classified as psychrophiles and were isolated, for example, in Antarctica [30]. The species *B. aurantiaca* has been shown to play a role in the biotechnological production of γ-decalactone with a peach flavor [31].

The sequence analysis of the A-043 isolate showed 100% similarity to the sequences of *Sporobolomyces ruberrimus* and *Sporidiobolus metaroseus* strains. Therefore, classical physiological tests were carried out, which showed the significant similarity of the A-043 strain to the known strains of *S. ruberrimus*. The characteristic features of this strain included the inability to assimilate arabinose and lactic acid (Table 4). In addition, the strain was unable to reproduce sexually by producing ellipsoidal teliospores, which is characteristic of *S. metaroseus*. The species *S. ruberrimus* was first described by Yamasaki and Fujii in 1950[32] and found to synthesize carotenoids[33] and lipases [34].

**Table 4:** Physiological characteristics of *Sporobolomyces*, *Sporidiobolus*, *Cystofilobasidium*, and *Erythrobasidium* isolates

Assimilation	A-012	A-038	A-043	A-044	A-051	A-056	A-058	A-076	A-079	A-104	S. ruberrimus*	S. metaroseus*	S. pararoseus*	*S. patagonicus* **	*C. macerans* *	C. infirmominiatum*	E. hasegawianum*
Glucose	+	+	+	+	+	+	+	+	+	+	+	+	+	+	+	+	+
D-Xylose	–	+	–	–	+	+	s	–	+	+	–	v	v	–	+	s	+
Galactose	+	+	+	+	+	+	+	–	–	+	l	+	+	l	+/s/w	v	s
L-Arabinose	+	–	–	–	+	s	+	–	+	s	–	–	v	–	+	+	+
L-Rhamnose	–	–	–	–	–	–	–	–	+	–	–	–	–	n	–	+	–
Fructose	+	+	+	+	+	+	+	+	+	+	n	n	n	n	n	n	n
Maltose	+	+	s	+	+	+	+	+	+	+	+	+	+	v	+	+	w
Saccharose	+	+	+	+	+	+	+	+	+	+	+	+	+	+	+	+	+
Lactose	–	–	–	–	–	–	–	–	–	–	–	–	–	–	v	v	–
Glycerol	+	+	+	+	+	s	+	+	s	+	l	+/s	+	l	s	s	+
D-Mannitol	+	+	+	+	+	+	+	+	+	+	l	+	+	l	+	+	+
Trehalose	+	+	s	+	+	+	+	+	-	+	+	+	+	v	+	s	+
Xylitol	+	+	–	+	–	+	+	s	+	s	n	–	s	n	+	+	n
Erythritol	–	–	–	–	–	+	–	–	–	–	–	–	–	–	+	–	–
Inulin	–	–	–	–	–	–	–	–	–	–	–	–	–	–	–	–	–
DL-Lactate	s	s	–	+	s	s	+	–	+	–	–	+	v	–	+/s	+	s
L-Tartaric acid	–	–	–	–	–	–	–	–	+	–	n	–	–	n	–	+	–
Citrate	+	+	+	+	+	+	–	+	+	+	l	+	+	–	+	+	+
Ethanol	+	+	+	+	–	+	+	+	+	+	+	+	+	–	+	+	–
Methanol	–	–	–	–	–	–	–	–	–	–	–	–	–	n	–	–	–
Raffinose	+	+	+	+	–	+	+	+	+	+	+	+	+	+	+	+	–
Nitrates	–	–	+	+	+	+	–	+	+	–	+	+	–	+	+	+	+
Vanillic acid	+	+	+	+	–	–	+	+	–	+	n	+	+	n	–	–	–
Gallic acid	–	–	–	–	–	–	–	–	–	–	n	–	–	n	–	–	–
Vitamins-free	+	+	+	+	–	–	+	+	–	+	+	+	+	+	–	–	–
Growth at 35 °C	–	–	–	–	–	–	–	–	–	–	–	–	–	–	–	–	–

*Typical strains from [8]; +, positive; l, latent (rapid development of a positive reaction after a lag period); s, positive but slow; w. weak; ws, weak and slow; lw, latent but weak (rapid development of a weak reaction after a lag period); v, variable; –, negative; n, no data

The obtained nucleotide sequence indicated that the A-044 strain belonged to *S. roseus* or *S. metaroseus* (Table 2). These species are very closely related; therefore, in 2008, it was assumed that *S. metaroseus* is a teleomorphic form of *S. roseus* [35]. All examined physiological features of the A-044 isolate corresponded to those described in “The Yeast—A Taxonomic Study” for typical representatives of *S. metaroseus* (anam. *S. roseus*) (Table 4). The A-044 strain showed no ability to produce teliospores on corn meal agar. Due to its inability to reproduce, this strain has been classified under *S. roseus* which has the ability to biosynthesize carotenoids [36] and aspartic protease [37], as well as take part in the transformation of wood lignins into technical lignins [38].

The isolates A-012, A-038, A-058, and A-104 were identified as *Sporidiobolus pararoseus* (Table 2). A characteristic feature of this yeast that distinguishes it from *S. metaroseus* and a significant number of *Sporobolomyces* yeasts is its inability to assimilate nitrates. On a solid medium, the strains of *S. pararoseus* produced ballistoconidia, which resulted in a mirror image of the colony in the upper Petri dish. All physiological tests of these isolates were consistent with the identification key of [8] for typical representatives of *S. pararoseus* (Table 4). Some *S. pararoseus* strains can be used in biocontrol [39] and in the production of exopolysaccharides [40], carotenoids, lipids [41], and enzymes for the synthesis of wine aromas [42].

Sequence analysis showed that the A-076 strain belonged to *S. pararoseus* or *Sporobolomyces* sp. with a 99.85% probability and to *Sporobolomyces patagonicus* with a 99.25% probability (Table 2). However, the membership of this isolate to *S. patagonicus* was excluded based on physiological tests. The name *patagonicus* was first proposed by Libkind et al. in 2005 for yeast strains isolated from aquatic environments in Patagonia (Argentina) [43]. So far, only two strains of this species (CBS 9657 and 9658) have been deposited in the collection of the Westerdijk Fungal Biodiversity Institute, which proves that these microorganisms are limited to specific regions of the world. The A-076 strain did not show similarity to *S. pararoseus*, as it could not assimilate galactose or nitrates (Table 4).

The strain A-056 was identified as *Cystofilobasidium macerans*, and strain A-079 as *Cystofilobasidium infirmominiatum* (Table 2). The genus *Cystofilobasidium* was first described by Oberwinkler in 1983 [44]. Originally, two species, namely *C. bisporidiis* and *C. capitatum*, were included in this genus, and later *C. infirmominiatum* and *C. ferigula* were added [45]. In 2009, Libkind et al. proposed to include two more species, *C. lacus-mascardi* and *C. macerans*, of which the last one is the sexual form of *Cryptocoocus macerans* [46]. The A-056 and A-079 strains showed all the morphological and physiological characteristics that are described as typical for the representatives of this species in ‘The Yeast – A Taxonomic Study’. So far, the biotechnological use of *Cystofilobasidium* yeasts has not been well described in the literature. These yeasts can synthesize carotenoids and polygalacturonase [47].

The A-051 strain was isolated from air and identified as *Erythrobasidium hasegawianum* (Table 2). The identification of selected physiological characteristics of this strain confirmed its genus and species (Table 4). The genus *Erythrobasidium* was created by Hamamoto, Sugiyama, and Komagata in 1988 and included only one species, which was previously classified as *Rhodotorula hasegawae* [48]. In 2015, Wang et al. included two more species (*E. elongatum* and *E. yunnanense*) which were previously classified in the genus *Sporobolomyces* [21]. The yeast *Erythrobasidium* is not widely distributed in the environment and has only been isolated from some sources, including the waters of the Calderone glacier (Italy) [49]. The potential biotechnological uses of *Erythrobasidium* have not been described so far.

Strain A-061, isolated from blueberry fruit, was identified as *Rhodotorula taiwanensis* (Tables 2 and 5). A yeast strain of this species was first isolated by Huang et al. in 2011 from the stem of the *Artemisia argyi* plant in Taiwan [50]. Since then, this yeast has also been isolated from the leaves of *Celtis bungeana* Bl. plants [51], sugarcane (*Saccharum officinarum* Linn.) [52] and traditional starter culture of Assam [53]. A characteristic feature of *Rhodotorula taiwanensis* yeasts is the lack of the ability to produce ballistoconidia or sexual reproduction, as well as the assimilation of ethanol and nitrites [50]. It was found that yeast strains belonging to this species can be a source of lipids for the production of biodiesel [51], biosurfactants [54] and carotenoids, including β-Cryptoxanthin [53], and also conduct the process of aluminum detoxification [55].

**Table 5:** Physiological characteristics of *Rhodotorula graminis*, *Rhodotorula glutinis*, *Rhodotorula diobovata*, *Rhodotorula taiwanensis, Rhodotorula toruloides* and *Rhodotorula* sp. isolates

Assimilation	A-020	A-031	A-037	A-048	A-055	A-061	A-064	A-087	A-088	A-101	A-106	A-111	R. graminis *	*R.* glutinis *	R. diobovata*	R. toruloides *	R. taiwanensis **
Glucose	+	+	+	+	+	+	+	+	+	+	+	+	+	+	+	+	+
D-Xylose	s	s	+	+	s	+	+	+	s	+	+	+	+	s	+	+	+
Galactose	+	+	+	+	+	+	+	+	+	+	+	+	+	+	+	+/s	+
L-Arabinose	+	+	+	+	+	+	+	+	s	+	–	+	+/w	+	v	+	+
L-Rhamnose	+	–	+	–	–	–	+	–	–	–	+	–	+	–	v	–	–
Fructose	+	+	+	+	+	+	+	+	+	+	+	–	+	n	+	n	n
Maltose	–	+	–	+	+	+	+	–	+	+	+	–	–	+	+	+	+
Saccharose	+	+	+	+	+	+	+	+	+	+	+	+	+	+	+	+	+
Lactose	–	–	–	–	–	–	–	–	–	–	–	–	–	–	–	–	–
Glycerol	+	+	+	+	+	+	+	+	+	+	s	+	+	+	+	+	+
D-Mannitol	+	+	+	w	+	+	+	+	+	+	s	+	+	+	+	+	+
Trehalose	s	s	+	w	+	+	+	+	s	+	+	+	+	+	+	+	+
Xylitol	–	+	–	+	s	+	–	+	+	+	–	–	–/s	+	+	+	+
Erythritol	–	–	–	–	–	–	+	–	–	–	–	–	–	–	–	–	–
Inulin	–	–	–	–	–	–	–	–	–	–	–	–	–	–	+/s	–	–
DL-Lactate	–	–	–	–	–	–	+	–	–	–	–	–	–	–	–	v	–
L-Tartaric acid	–	+	–	–	+	–	+	–	+	+	+	–	–	+	–	–	n
Citrate	+	+	+	+	+	+	s	+	+	+	+	+	+	+	+	+/s	+
Ethanol	+	+	+	+	+	–	+	+	+	+	s	–	+	+	+	+	–
Methanol	–	–	–	–	–	–	–	–	–	–	–	–	–	–	–	–	–
Raffinose	+	+	+	+	+	+	+	+	+	s	+	+	+	+/s	+	+	+
Nitrates	+	+	+	+	+	+	+	+	+	+	+	+	+	+	+	+	+
Vanillic acid	+	+	+	+	+	+	+	+	+	+	+	+	+	+	+	+	n
Gallic acid	–	–	–	+	–	–	–	+	–	–	–	+	–	–	+	+	n
Glucuronic acid	–	–	–	–	–	–	–	+	–	–	–	–	–	–	n	+	n
Vitamins-free	+	+	+	+	+	–	+	+	+	+	+	+	+	+	+	+	n
Growth at 35 °C	–	–	–	+	–	+	–	+	–	–	–	+	–	–	+	+	+

*Typical strains from The and [56]; ** [50]; +, positive; l, latent (rapid development of a positive reaction after a lag period); s, positive but slow; w, weak; ws, weak and slow; lw, latent but weak (rapid development of a weak reaction after a lag period); v, variable; –, negative; n, no data

The isolates A-087 and A-111 were identified as belonging to *R. toruloides*. The results of the biochemical tests were consistent with the identification key of Kurtzman et al. (2011). Only the A-111 strain could not grow in the medium lacking vitamins (Table 5). The species *R. toruloides* is classified under the family Sporidiobolaceae, the order Sporidiobolales, the class Microbotryomycetes, and the class Basidiomycota. Some edible mushrooms, rusts, and smuts are also included in this group. Originally, *R. toruloides* was classified as *Rhodosporidium toruloides*, but in 2015 Wang and colleagues proposed to include this species as well as *Rhodosporidium diobovatum*, *Rhodosporidium babjevae*, *Rhodosporidium kratochvilovae*, *Rhodosporidium paludigenum*, and *Rhodosporidium sphaerocarpum* in the genus *Rhodotorula*. The yeast *R. toruloides* can biosynthesize lipids and carotenoids [57–59].

Sequence analysis of the the remaining red yeast strains revealed that they possibly belonged mainly to *Rhodotorula glutinis*, *Rhodotorula graminis*, *Rhodotorula babjevae*, *Rhodotorula diobovata* or *Ustilentyloma graminis* (Table 2). Gadanho and Sampaio reported that *R. glutinis*, *R. babjevae*, and *R. graminis* show close phenetic and phylogenetic proximity and indicated various assimilation tests which allow distinguishing these species from one another. A characteristic feature of *R. glutinis* is its ability to assimilate L-arabinose, maltose, tartaric acid, and nitrates and inability to assimilate L-rhamnose, gallic acid, and lactates. The yeast *R. graminis* can metabolize both L-rhamnose and L-arabinose, and does not absorb maltose, gallic and tartaric acid, and lactate. The typical strains of *R. babjevae* can assimilate L-rhamnose, maltose, lactate, and tartaric acid. A characteristic feature of *R. diobovata* is that it can grow up to 35℃ and assimilate L-arabinose, maltose, and gallic acid [56]. *Rhodotorula hordea* was first included in the genus *Ustilentyloma* by Wang et al. in 2015 under the name *Ustilentyloma graminis* [21]. The characteristic features of this yeast are the yellow-cream color of the colony, inability to assimilate L-rhamnose, L-arabinose, xylose, ethanol, xylitol, vanillic acid, gallic acid, and ability to grow in a nutrient-free medium [8]. None of the yeast isolates tested in this study showed these features (Tables 5 and 6).

**Table 6:** Physiological characteristics of *Rhodotorula babjevae* and isolates

Assimilation	A-009	A-010	A-013	A-014	A-026	A-030	A-036	A-041	A-054	A-068	A-070	A-086	A-095	A-099	A-100	A-102	A-103	A-110	R. babjevae*
Glucose	+	+	+	+	+	+	+	+	+	+	+	+	+	+	+	+	+	+	+
D-Xylose	+	+	+	+	+	+	+	w	+	+	+	+	+	+	+	+	+	+	+
Galactose	+	+	+	+	+	+	+	+	+	+	+	+	+	+	+	+	+	+	+
L-Arabinose	–	–	–	–	–	–	–	–	–	–	–	–	–	–	–	–	–	–	–
L-Rhamnose	+	+	+	+	+	+	+	+	+	+	+	+	+	+	+	+	+	+	+
Fructose	+	+	+	+	+	+	+	+	+	+	+	+	+	+	+	+	+	+	n
Maltose	+	+	+	+	+	+	+	+	+	+	+	+	+	+	+	+	+	+	+
Saccharose	+	+	+	+	+	+	+	+	+	+	+	+	+	+	+	+	+	+	+
Lactose	–	–	–	–	–	–	–	–	–	–	–	–	–	–	–	–	–	–	–
Glycerol	+	+	+	+	+	+	+	+	+	+	+	+	+	+	+	+	+	+	+
D-Mannitol	+	+	+	+	+	+	+	+	+	+	+	+	+	+	+	+	+	+	+
Trehalose	+	+	+	+	s	+	+	–	s	+	+	s	+	+	+	s	+	s	+
Xylitol	+	+	+	+	s	+	+	–	+	+	+	+	+	+	+	+	+	+	+
Erythritol	–	–	–	–	–	–	–	–	–	–	–	–	–	–	–	–	–	–	–
Inulin	–	–	s	–	–	–	s	–	–	–	s	–	–	–	–	s	–	–	s/–
DL-Lactate	+	s	+	+	+	+	s	s	+	+	+	s	+	+	+	+	+	s	+/s
L-Tartaric acid	+	+	+	–	–	–	+	+	+	+	+	–	+	+	+	+	+	–	v
Citrate	+	+	+	+	+	+	+	+	+	+	+	+	+	+	+	+	+	+	+
Ethanol	+	+	+	+	+	+	+	+	+	+	+	+	+	+	+	+	+	+	+
Methanol	–	–	–	–	–	–	–	–	–	–	–	–	–	–	–	–	–	–	–
Raffinose	+	+	+	+	+	+	+	+	+	+	+	+	+	+	+	+	+	+	+
Nitrates	+	+	+	+	+	+	+	+	+	+	+	+	+	+	+	+	+	+	+
Vanillic acid	+	+	+	+	+	+	+	+	+	+	+	+	+	+	+	+	+	+	+
Gallic acid	–	–	–	–	–	–	–	–	–	–	–	–	–	–	–	–	–	–	–
Glucuronic acid	+	+	+	+	+	+	+	+	+	+	+	+	+	+	+	+	+	+	+
**Vitamins-free**	+	+	+	+	+	+	+	+	+	+	+	+	+	+	+	+	+	+	+
**Growth at 35℃**	–	–	–	–	–	–	–	–	–	–	–	–	–	–	–	–	–	–	–

*Typical strains from [8] and [56]; +, positive; l, latent (rapid development of a positive reaction after a lag period); s, positive but slow; w, weak; ws, weak and slow; lw, latent but weak (rapid development of a weak reaction after a lag period); v, variable; –, negative; n, no data

Only the A-048 isolate (Table 5) showed all characteristics of *R. diobovata*. It was the only tested strain to assimilate gallic acid. Another characteristic feature of this strain was the ability to grow up to 35℃. In 1970, Newell and Hunter isolated the first strain of the yeast *Rhodosporidium diobovatum* (teleomorphic form) from seawater collected in South Florida [60]. This yeast was also isolated from deep-sea sediments of the eastern Pacific Ocean [61] and cotton grown in Egypt [62]. The yeast *R. diobovata* may have great biotechnological potential. It is classified as oleaginous and can synthesize and accumulate mainly triacylglycerols. It also has the ability to use waste carbon and nitrogen sources, which reduces the environmental burden of industrial waste [63]. Some strains of *R. diobovata* can effectively remove inorganic forms of nitrogen from the environment and thus play an important role in bioremediation and wastewater treatment [64].

As reported by Gadanho and Sampaio, a characteristic feature of *R. babjevae* is the ability to assimilate D-glucuronate, which cannot be found in the species *R. glutinis* and *R. graminis* and thus allows easy differentiation between them [56]. In this study, the D-glucuronate-assimilating capacity was exhibited by strains A-009, A-010, A-013, A-014, A-026, A-030, A-036, A-041, A-054, A-068, A-070, A-086, A-095, A-099, A-100, A-102, A-103 and A-110 (Table 6). The analysis of other physiological features confirmed that these strains belonged to *R. babjevae*. Gadanho and Sampaio also reported that *R. babjevae* is widespread in nature and some of the strains previously identified as *R. glutinis* may actually belong to *R. babjevae*. The possibilities of using these microorganisms in various industries have been widely investigated by researchers. The most important areas of research include the production of biosurfactants [65], carotenoids [66], microbial lipids [67], and glycolipids [68].

The typical features of *R. glutinis* were shown by isolates A-031, A-055, A-088 and A-101. These strains were able to assimilate L-arabinose, maltose, tartaric acid, and nitrates, and failed to absorb L-rhamnose and gallic acid (Table 5). The yeast *R. glutinis* was first isolated from sour cream in 1850. Currently, this species is considered typical of the entire genus *Rhodotorula* [8]. This yeast can be used as a source of SCO [69], carotenoids [70], α-L-arabinofuranosidase [71], PAL [72], and lipases [73].

Strains A-020 and A-037 were identified as belonging to *R. graminis* (Table 2) because they could not assimilate maltose, tartaric acid, and lactic acid, which allowed distinguishing from *R. babjevae* and *R. glutinis* (Table 5). The species *R. graminis* belongs to the group of *R. glutinis* sensu stricto together with *R. glutinis* and *R. babjevae*; however, it is much less frequently isolated compared to these species [56]. In 1958, Margaret di Menna isolated the first strain of *R. graminis* from the leaf surface of pasture grasses on the North Island of New Zealand [74]. Since then, the yeasts of this species have been considered as a valuable source of lipids [75], L-phenylalanine ammonia-lyase (PAL) [76] and carotenoids [77].

Strains A-064 and A-106 were identified as belonging only to the genus *Rhodotorula* because the assimilation profiles of the tested compounds did not allow for their unambiguous classification into *R. graminis*, *R. babjevae*, or *R. glutinis* species.

### Summary

Red yeasts are representatives of natural microflora found in different environments in Poland. In this study, a total of 114 red yeast strains were isolated from plant organisms (e.g., flowers, leaves) and environments (e.g., air). All isolated strains which had been pre-selected by formation of colored colonies, synthesized carotenoid pigments. The total content of carotenoids in cellular biomass ranged from 13.88 to 406.50 µg/g. Torulene is a compound that occurs in the highest amounts in yeast cells. Among the yeast isolates, torulene accounted for more than 50% of the total carotenoid pool in 49 strains. The largest number of strains belonged to the genera *Rhodotorula* (37), *Sporidiobolus* (4), *Sporobolomyces* (3), *Cystofilobasidium* (2), *Buckleyzyma* (1) and *Erythrobasidium* (1).

## Data Availability

The data presented in this study are available on request from the corresponding author.
